# Convective Flow of Sisko Fluid over a Bidirectional Stretching Surface

**DOI:** 10.1371/journal.pone.0130342

**Published:** 2015-06-25

**Authors:** Asif Munir, Azeem Shahzad, Masood Khan

**Affiliations:** 1 Department of Mathematics, Quaid-i-Azam University, Islamabad, Pakistan; 2 Basic Science Department, UET, Taxila, Pakistan; Northwestern Polytechnical University, CHINA

## Abstract

The present investigation focuses the flow and heat transfer characteristics of the steady three-dimensional Sisko fluid driven by a bidirectional stretching sheet. The modeled partial differential equations are reduced to coupled ordinary differential equations by a suitable transformation. The resulting equations are solved numerically by the shooting method using adaptive Runge Kutta algorithm in combination with Newton's method in the domain [0,∞). The numerical results for the velocity and temperature fields are graphically presented and effects of the relevant parameters are discussed in detail. Moreover, the skin-friction coefficient and local Nusselt number for different values of the power-law index and stretching ratio parameter are presented through tabulated data. The numerical results are also verified with the results obtained analytically by the homotopy analysis method (HAM). Additionally, the results are validated with previously published pertinent literature as a limiting case of the problem.

## Introduction

The phenomena of momentum and heat transfer in boundary layer flow over a flat heated surface are experienced widely in industrial engineering applications. The momentum and heat transfer due to a heated stretching surface have gained considerable attention because of their practical importance in diverse engineering disciplines. Plastic and rubber sheets are manufactured by this process, where it is often necessary to blow a gaseous medium through the not yet solidified material. Further example that belongs to the above class of problems is the cooling of a large metallic plate in a bath, which may be an electrolytic [1]. The quality of finished product is strongly dependent upon the final cooling of the product. Various aspects of such problems, including unidirectional and bidirectional stretching surface, have been the focal point of many theoretical researchers. Some previous works regarding bidirectional stretching surface was carried out by Wang [2], who presented exact similar solutions for a three-dimensional flow due to stretching of sheet in two lateral directions. Later on, Ariel [3] addressed this problem by finding the approximate analytical solutions using the homotopy perturbation method. Liu and Andersson [4] also explored numerically the heat transfer characteristics of fluid, when the sheet is stretched in two lateral directions with variable thermal boundary conditions. Lakshmisha *et al*. [5] obtained numerical solutions of unsteady three-dimensional boundary layer with constant temperature and heat flux thermal boundary conditions. Ahmad *et al*. [6] provided the approximate analytical solutions of the problem for heat transfer in hydromagnetic flow induced by bidirectional stretching sheet in porous medium.

All of the abovementioned studies lie within the domain of a Newtonian fluid. There is a vast utility of non-Newtonian fluids in industrial sector such as pharmaceutical, polymer, personal care products and so forth. Various researchers explored the flow of non-Newtonian fluids due to bidirectional stretching surface, including Gorla *et al*. [1] who addressed the three dimensional flow of a power-law fluid over a bidirectional stretching surface. Recently, Nadeem *et al*. [7] analyzed the MHD three-dimensional Casson fluid flow past a porous linearly stretching sheet. Shehzad *et al*. [8,9] also studied the three-dimensional flow of Maxwell and Oldroyd-B fluids over bi-dimensional stretching surface. Shehzad *et al*. [10] further explored the three-dimensional flow of Jeffery fluid including the effects of magneto-hydrodynamics and Newtonian heating.

From the foregoing information, it is much clear that no attempt has been made so far to study the three dimensional boundary layer flows and heat transfer characteristics of Sisko fluid over a bidirectional stretching surface. This paper is thus concerned with the boundary layer flow of Sisko fluid over an isothermal bidirectional surface and the effects different related parameters on velocities and temperature field are explored in depth.

## Physical model and mathematical formulation

### Rheological model

In the current study we consider time independent non-Newtonian fluid that follows Sisko rheological model. The Cauchy stress tensor **T** for such fluid is given by

T=−pI+S, (1)

where **S** is the extra stress tensor given by

$$S=\left[a+b{\left|\sqrt{\frac{1}{2}tr(A_{1}^{2})}\right|}^{n-1}\right]A_{1},$$ (2)

in which *n* > 0, *a* and *b* are the physical constants different for different fluid, **A**_1_ = (*grad* **V**) + (*gard* **V**)^T^ the first Rivlin-Erickson tensor with **V** the velocity vector and T stands for transpose.

### Boundary layer equations

The continuity and momentum equations for the steady flow of an incompressible Sisko fluid with the velocity field **V** = [*u*(*x*, *y*, *z*), *v*(*x*, *y*, *z*), *w*(*x*, *y*, *z*)] are stated as follows:

$$\frac{\partial u}{\partial x}+\frac{\partial v}{\partial y}+\frac{\partial w}{\partial z}=0,$$ (3)

$$\begin{array}{c} \rho \left(u\frac{\partial u}{\partial x}+v\frac{\partial u}{\partial y}+w\frac{\partial u}{\partial z}\right)=-\frac{\partial p}{\partial x}+a\left(\frac{\partial^{2}u}{\partial x^{2}}+\frac{\partial^{2}u}{\partial y^{2}}+\frac{\partial^{2}u}{\partial z^{2}}\right)+2b\frac{\partial }{\partial x}\left[\frac{\partial u}{\partial x}{\left|\sqrt{\frac{1}{2}trA_{1}^{2}}\right|}^{n-1}\right]+\\ b\frac{\partial }{\partial y}\left[\left(\frac{\partial u}{\partial y}+\frac{\partial v}{\partial x}\right){\left|\sqrt{\frac{1}{2}trA_{1}^{2}}\right|}^{n-1}\right]+b\frac{\partial }{\partial z}\left[\left(\frac{\partial u}{\partial z}+\frac{\partial w}{\partial x}\right){\left|\sqrt{\frac{1}{2}trA_{1}^{2}}\right|}^{n-1}\right], \end{array}$$ (4)

$$\begin{array}{c} \rho \left(u\frac{\partial v}{\partial x}+v\frac{\partial v}{\partial y}+w\frac{\partial v}{\partial z}\right)=-\frac{\partial p}{\partial y}+a\left(\frac{\partial^{2}v}{\partial x^{2}}+\frac{\partial^{2}v}{\partial y^{2}}+\frac{\partial^{2}v}{\partial z^{2}}\right)+b\frac{\partial }{\partial x}\left[\left(\frac{\partial u}{\partial y}+\frac{\partial v}{\partial x}\right){\left|\sqrt{\frac{1}{2}trA_{1}^{2}}\right|}^{n-1}\right]+\\ 2b\frac{\partial }{\partial y}\left[\left(\frac{\partial v}{\partial y}\right){\left|\sqrt{\frac{1}{2}trA_{1}^{2}}\right|}^{n-1}\right]+b\frac{\partial }{\partial z}\left[\left(\frac{\partial v}{\partial z}+\frac{\partial w}{\partial y}\right){\left|\sqrt{\frac{1}{2}trA_{1}^{2}}\right|}^{n-1}\right], \end{array}$$ (5)

$$\begin{array}{c} \rho \left(u\frac{\partial w}{\partial x}+v\frac{\partial w}{\partial y}+w\frac{\partial w}{\partial z}\right)=-\frac{\partial p}{\partial z}+a\left(\frac{\partial^{2}w}{\partial x^{2}}+\frac{\partial^{2}w}{\partial y^{2}}+\frac{\partial^{2}w}{\partial z^{2}}\right)+b\frac{\partial }{\partial x}\left[\left(\frac{\partial u}{\partial z}+\frac{\partial w}{\partial x}\right){\left|\sqrt{\frac{1}{2}trA_{1}^{2}}\right|}^{n-1}\right]+\\ b\frac{\partial }{\partial y}\left[\left(\frac{\partial v}{\partial z}+\frac{\partial w}{\partial y}\right){\left|\sqrt{\frac{1}{2}trA_{1}^{2}}\right|}^{n-1}\right]+2b\frac{\partial }{\partial z}\left[\frac{\partial w}{\partial z}{\left|\sqrt{\frac{1}{2}trA_{1}^{2}}\right|}^{n-1}\right], \end{array}$$ (6)

where

$$\frac{1}{2}tr\left(A_{1}^{2}\right)=2{\left(\frac{\partial u}{\partial x}\right)}^{2}+2{\left(\frac{\partial v}{\partial y}\right)}^{2}+2{\left(\frac{\partial w}{\partial z}\right)}^{2}+{\left(\frac{\partial u}{\partial y}+\frac{\partial v}{\partial x}\right)}^{2}+{\left(\frac{\partial u}{\partial z}+\frac{\partial w}{\partial x}\right)}^{2}+{\left(\frac{\partial v}{\partial z}+\frac{\partial w}{\partial y}\right)}^{2}.$$ (7)

Defining the dimensionless variables and parameters as

$$u^{*}=\frac{u}{U},\;v^{*}=\frac{v}{U},\;w^{*}=\frac{wL}{U\delta },\;x^{*}=\frac{x}{L},\;y^{*}=\frac{y}{L},\;z^{*}=\frac{z}{\delta },\;\text{and}\;p^{*}=\frac{p}{\rho U^{2}},$$ (8)

where *L* is the characteristic length and *U* the characteristic velocity. Noting that inertial and viscous forces are of the same order of magnitude within the boundary layer, taking $\frac{a/\rho }{LU}{\left(\frac{L}{\delta }\right)}^{2}=O(1),$ $\frac{b/\rho }{L^{n}U^{2-n}}{\left(\frac{L}{\delta }\right)}^{n+1}=O(1)$, and under the assumption of large Reynolds number, Eqs (3)–(7), in terms of dimensionless variables, asymptotically can be casted as

$$\frac{\partial u^{*}}{\partial x^{*}}+\frac{\partial v^{*}}{\partial y^{*}}+\frac{\partial w^{*}}{\partial z^{*}}=0,$$ (9)

$$u^{*}\frac{\partial u^{*}}{\partial x^{*}}+v^{*}\frac{\partial u^{*}}{\partial y^{*}}+w^{*}\frac{\partial u^{*}}{\partial z^{*}}=-\frac{\partial p^{*}}{\partial x^{*}}+\frac{\partial^{2}u^{*}}{\partial z^{*}{}^{2}}+\frac{\partial }{\partial z^{*}}\left[\frac{\partial u^{*}}{\partial z^{*}}{\left|\frac{\partial u^{*}}{\partial z^{*}}\right|}^{n-1}\right],$$ (10)

$$u^{*}\frac{\partial v^{*}}{\partial x^{*}}+v^{*}\frac{\partial v^{*}}{\partial y^{*}}+w^{*}\frac{\partial v^{*}}{\partial z^{*}}=-\frac{\partial p^{*}}{\partial y^{*}}+\frac{\partial^{2}v^{*}}{\partial z^{*}{}^{2}}+\frac{\partial }{\partial z^{*}}\left[\frac{\partial v^{*}}{\partial z^{*}}{\left|\frac{\partial u^{*}}{\partial z^{*}}\right|}^{n-1}\right],$$ (11)

$$0=-\frac{\partial p^{*}}{\partial z^{*}},$$ (12)

Eqs (9)–(11) in dimensional form simplify as

$$\frac{\partial u}{\partial x}+\frac{\partial v}{\partial y}+\frac{\partial w}{\partial z}=0,$$ (13)

$$\rho \left(u\frac{\partial u}{\partial x}+v\frac{\partial u}{\partial y}+w\frac{\partial u}{\partial z}\right)=-\frac{\partial p}{\partial x}+a\frac{\partial^{2}u}{\partial z^{2}}+b\frac{\partial }{\partial z}\left({\left|\frac{\partial u}{\partial z}\right|}^{n-1}\frac{\partial u}{\partial z}\right),$$ (14)

$$\rho \left(u\frac{\partial v}{\partial x}+v\frac{\partial v}{\partial y}+w\frac{\partial v}{\partial z}\right)=-\frac{\partial p}{\partial y}+a\frac{\partial^{2}v}{\partial z^{2}}+b\frac{\partial }{\partial z}\left({\left|\frac{\partial u}{\partial z}\right|}^{n-1}\frac{\partial v}{\partial z}\right).$$ (15)

### Governing equations and boundary conditions

Considering the three-dimensional steady, laminar and incompressible flow of fluid obeying the Sisko model which occupies space *z* > 0. The fluid is set into motion by an elastic flat sheet in the plane *z* = 0, kept at a constant temperature, which is being continuously stretched with linear velocities *cx* and *dy* in the *x-* and *y-* directions, respectively. The constants *c* and *d* are positive real numbers relating to stretching of the sheet. The ambient temperature far away from the sheet is uniform and taken as *T*_∞_. The continuity, momentum and energy equations governing the steady three-dimensional flow of Sisko fluid, approximated by boundary-layer theory are

$$\frac{\partial u}{\partial x}+\frac{\partial v}{\partial y}+\frac{\partial w}{\partial z}=0,$$ (16)

$$\rho \left(u\frac{\partial u}{\partial x}+v\frac{\partial u}{\partial y}+w\frac{\partial u}{\partial z}\right)=a\frac{\partial^{2}u}{\partial z^{2}}-b\frac{\partial }{\partial z}{\left(-\frac{\partial u}{\partial z}\right)}^{n},$$ (17)

$$\rho \left(u\frac{\partial v}{\partial x}+v\frac{\partial v}{\partial y}+w\frac{\partial v}{\partial z}\right)=a\frac{\partial^{2}v}{\partial z^{2}}+b\frac{\partial }{\partial z}{\left(-\frac{\partial u}{\partial z}\right)}^{n-1}\frac{\partial v}{\partial z},$$ (18)

$$u\frac{\partial T}{\partial x}+v\frac{\partial T}{\partial y}+w\frac{\partial T}{\partial z}=\frac{\kappa }{\rho c_{p}}\frac{\partial^{2}T}{\partial z^{2}}.$$ (19)

Here (*u*, *v*, *w*) are unknown velocity components *x*, *y* and *z* directions, *T* the temperature, *ρ* the fluid density, *c*_*p*_ the specific heat of fluid at constant pressure and *κ* the thermal conductivity.

Eqs (16)–(19) are subjected to the following boundary conditions

$$u=U_{w}(x)=cx,\;v=V_{w}(y)=dy,\;w=0,\;T=T_{w}\;\text{at}\;z=0,$$ (20)

$$u\to 0,\;v\to 0,\;w\to 0,\;T\to T_{\infty }\;\text{as}\;z\to \infty .$$ (21)

### Transformed problem

The governing coupled partial differential Eqs (17)–(19) are transformed to coupled ordinary differential equations by introducing transformation variables

$$u=cxf\prime (\eta ),\;v=dyg\prime (\eta ),\;w=-c{\left(\frac{c^{n-2}}{\rho /b}\right)}^{1/(n+1)}\left(\frac{2n}{n+1}f+\frac{1-n}{1+n}\eta f\prime +g\right)x^{\left(n-1\right)/\left(n+1\right)},$$

$$\theta \left(\eta \right)=\frac{T(x,y,z)-T_{\infty }}{T_{w}-T_{\infty }},\;\eta =z{\left(\frac{c^{2-n}}{b/\rho }\right)}^{1/n+1}x^{\left(1-n\right)/(1+n)}.$$ (22)

The momentum and heat transfer equations with the associated boundary conditions reduce to

$$Af\prime \prime \prime +n{\left(-f\prime \prime \right)}^{n-1}f\prime \prime \prime +\frac{2n}{n+1}ff\prime \prime -{\left(f\prime \right)}^{2}+gf\prime \prime =0,$$ (23)

$$Ag\prime \prime \prime +{\left(-f\prime \prime \right)}^{n-1}g\prime \prime \prime -\left(n-1\right)g\prime \prime f\prime \prime \prime {\left(-f\prime \prime \right)}^{n-2}+\frac{2n}{n+1}fg\prime \prime -{\left(g\prime \right)}^{2}+gg\prime \prime =0,$$ (24)

$$\theta \prime \prime +\text{Pr}\left(\frac{2n}{n+1}\right)f\theta \prime +\text{Pr}\;g\theta \prime =0,$$ (25)

f(0)+g(0)=0,f′(0)=1,g′(0)=d/c=α,θ(0)=1, (26)

f′(η)→0,g'(η)→0andθ(η)→0asη→∞, (27)

with no loss of generality we can set

f(0)=0andg(0)=0.

where the prime stand for differentiation with respect *η* and *α* is the stretching ratio parameter. We are considering the range 0 ≤ *α* ≤ 1, since for *α* > 1, the *x* and *y* axes are interchanged [2]. Further, Re_*a*_, Re_*b*_ are the local Reynolds number, *A* the material parameter of Sisko fluid and Pr the generalized Prandtl number, which are defined as

$${\text{Re}}_{a}=\frac{\rho xU}{a},\;{\text{Re}}_{b}=\frac{\rho x^{n}U^{2-n}}{b},\;A=\frac{{\text{Re}}_{b}^{\frac{2}{n+1}}}{{\text{Re}}_{a}}\;\text{and}\;\text{Pr}=\frac{xUR_{b}^{\frac{-2}{n+1}}}{\kappa /\rho c_{p}}.$$ (28)

Note that in the limit *α* → 0 the unidirectional case is obtained and motion of fluid is merely in *xz* plane, i.e. *g* and *g*′ in Eq (22) both are zero. When, *α* = 1, the stretching rate is same in the *x-* and *y*- directions and the flow is axisymmetric.

### Important physical parameters

The physical quantities of main interest are the skin-friction coefficient and the local Nusselt number.

#### The skin-friction coefficients

The skin coefficient is an important boundary layer characteristic, which is the dimensionless shear stress at the wall (*z* = 0). Thus the dimensionless skin friction coefficients along the *x-* and *y-* directions, respectively, are given by

$$C_{fx}=\frac{\tau_{xz}}{\frac{1}{2}\rho U_{w}^{2}}\;\text{and}\;C_{fy}=\frac{\tau_{yz}}{\frac{1}{2}\rho U_{w}^{2}},$$ (29)

where *τ*_*xz*_ and *τ*_yz_ are shear stresses along the *x-* and *y*- directions, respectively. These quantities in dimensionless form can be expressed as

$$\frac{1}{2}{\text{Re}}_{b}^{1/\left(n+1\right)}C_{fx}=Af\prime \prime \left(0\right)-{\left[-f\prime \prime \left(0\right)\right]}^{n},$$ (30)

$$\frac{1}{2}{\text{Re}}_{b}^{1/\left(n+1\right)}C_{fy}=\frac{V_{w}}{U_{w}}\left[Ag\prime \prime \left(0\right)+{\left[-f\prime \prime \left(0\right)\right]}^{n-1}g\prime \prime (0)\right].$$ (31)

#### The local Nusselt number

The local Nusselt number denoted by *Nu*_*x*_, giving the rate of heat transfer at the wall, is defined by

$$Nu_{x}={\frac{xq_{w}}{\kappa \left(T_{w}-T_{\infty }\right)}|}_{z=0},$$ (32)

where the wall heat flux defined by $q_{w}=-\kappa {\left(\frac{\partial T}{\partial z}\right)|}_{z=0}$ resulting in

$${\text{Re}}_{b}^{-1/n+1}\;Nu_{x}=-\theta \prime \left(0\right).$$ (33)

## Solution Methodologies

We are not able to find the exact analytical solution of the non-linear two point boundary value problem (23)-(27). Consequently, these equations are solved numerically by the shooting technique. The equations are written as a system of eight first order ordinary differential equations. The corresponding initial value problem is solved by adaptive Runge-Kutta method. The Newton’s iterative algorithm is used to assure quadratic convergence of iteration required to satisfy the boundary condition at infinity.

### Analytical solution method

Analytical results are sought using the homotopy analysis method (HAM) for certain values of parameters with a view to check the veracity of our numerical results. To apply the HAM, we selected the initial guesses agreeing the boundary data as

$$f_{0}=1-e^{-\eta },\;g_{0}=1-e^{-\eta }\;\text{and}\;\theta_{0}=e^{-\eta },$$ (34)

and auxiliary linear operators

$$L_{f}=f\prime \prime \prime -f\prime ,L_{g}=g\prime \prime \prime -g\prime \;\text{and}\;L_{\theta }=\theta \prime \prime -\theta ,$$ (35)

for the velocity and temperature fields, respectively.

The proper values of convergence control parameters ℏ_*f*_, ℏ_*g*_ and ℏ_*θ*_ assure the convergence of series solution. The optimal values of these parameters are chosen by minimizing the discrete squared residual error [11].

## Validation of the numerical results

**Fig 1:**
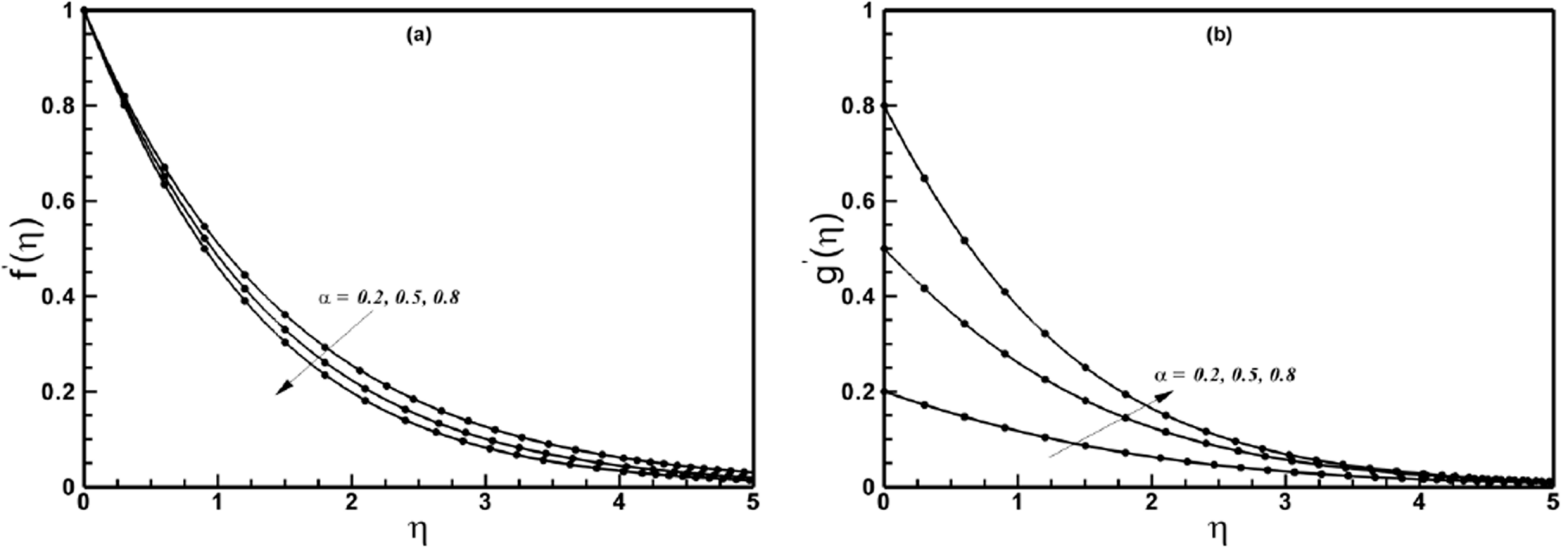
A comparison between the numerical and HAM results (solid lines numerical and solid circles HAM results) when *n* = 1.0 and *A* = 1.5 are fixed.

The validation of results is essential to check the credibility of the numerical solution methodology. The presently computed results are compared with the analytical results obtained by HAM, and an excellent correspondence is seen to exist between the two sets of data as shown in Fig 1A and 1B. Additionally, the results are also compared with previous published relevant literature (Table 1), as a special case of the problem and excellent agreement is observed in this case also.

**Table 1:** A comparison of the local skin friction coefficient and local Nusselt number when *n* = 1 and *A* = 0 are fixed.

	*α*	*f*″(0)	*g*″(0)	*θ*′(0)
Ref. [1]	0.00	-1.00000	0	-
Ref. [3]	0.00	-1.00000	0	-
Present study	0.00	-1.00000	0	-
Ref. [1]	0.25	-1.048813	-0.194564	-
Ref. [3]	0.25	-1.048813	-0.194565	-0.665933
Present study	0.25	-1.048818	-0.194567	-0.665939
Ref. [1]	0.50	-1.093097	-0.465205	-
Ref. [3]	0.50	-1.093096	-0.465206	-0.735334
Present study	0.50	-1.093098	-0.465207	-0.735336
Ref. [1]	0.75	-1.134485	-0.794622	-
Ref. [3]	0.75	-1.134486	-0.794619	-0.796472
Present study	0.75	-1.134487	-0.794619	-0.796472
Ref. [1]	1.00	-1.173720	-1.173720	-
Ref. [3]	1.00	-1.173721	-1.173721	-
Present study	1.00	-1.173721	-1.173721	-

## Results and Discussion

This article predominantly focuses the flow and heat transfer characteristics of Sisko fluid past a uniformly heated and bidirectional stretching surface. To grasp both the phenomena, Eqs (23)–(25) with the boundary conditions (26) and (27) are solved numerically for non-integer values of the power-law index *n* and results are presented graphically. The effects of various parameters like the power-law index *n*, material parameter *A* and stretching ratio parameter *α* are investigated for non-dimensional velocity components *f*′(*η*), *g*′(*η*) and temperature *θ*(*η*). Variation of the local skin friction coefficients and local Nusselt number are also observed for different values of *n* and *α*.

**Fig 2:**
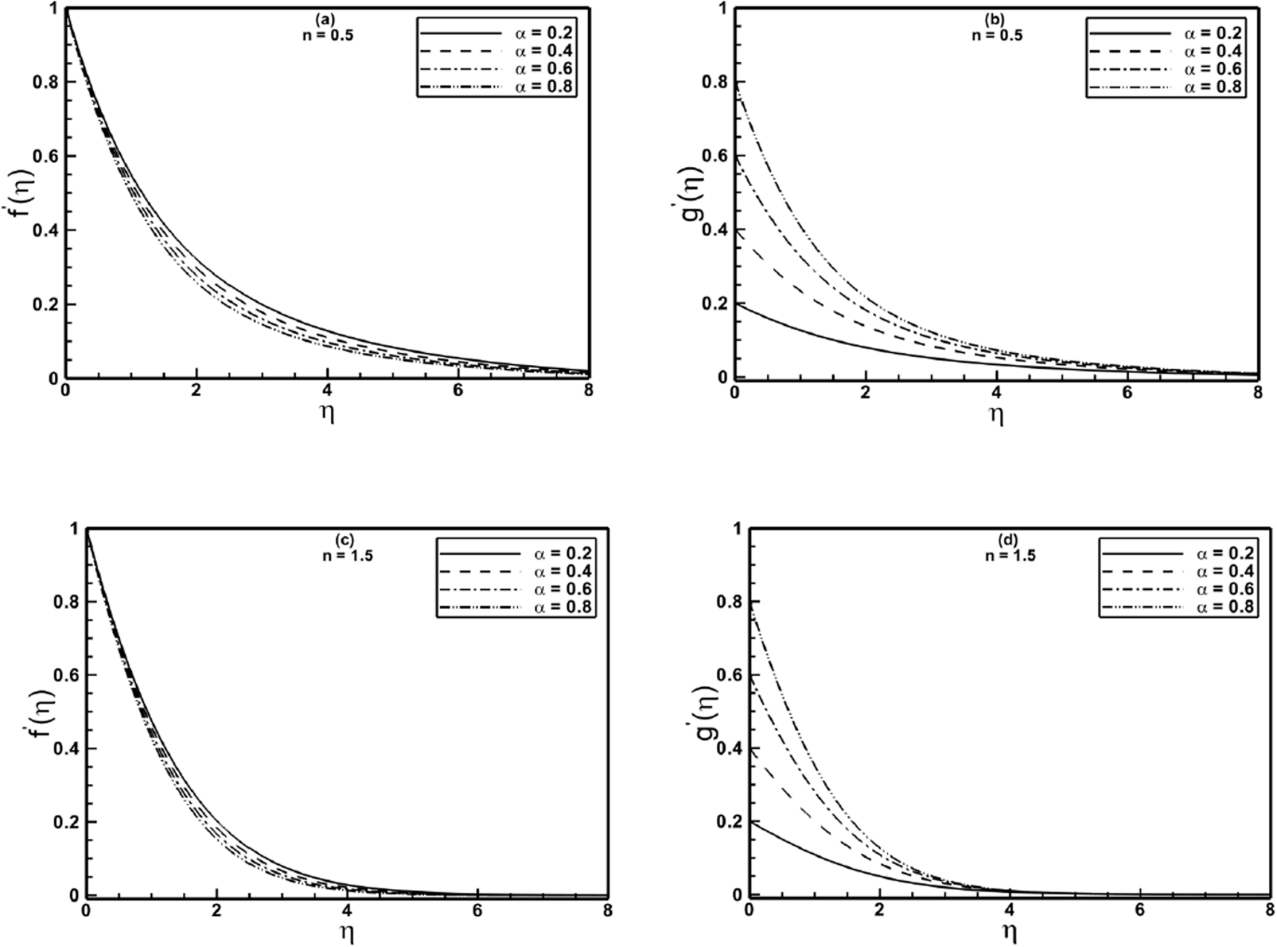
Profiles of the velocities *f*′(*η*) and *g*′(*η*) for different values of the stretching ratio parameter *α* when *A* = 1.5 is fixed.

Fig 2A–2F present the effect of the stretching ratio parameter *α* on the velocity components *f*′(*η*) and *g*′(*η*) for Sisko fluid with shear thinning (*n* = 0.5) and shear thickening (*n* = 1.5) properties. It is evident from Fig 2A that *f*′(*η*) decreases with increasing values of *α*, however, opposite behavior is observed for the *g*′(*η*) component of the velocity (Fig 2B). This behavior of these velocity components is due to the fact that augmentation in *α*, enhances the stretching rate in *y*- direction. A comparison of Fig 2A and 2B reveals that the stretching ratio parameter affects *g*′(*η*) component more pronouncedly. Fig 2C and 2D show the same qualitative trends but there is noticeable decrease in boundary layer thickness for shear thickening fluid as compared to that of shear thinning fluid, because lesser momentum is transferred between the stretching surface and still fluid, owing to increased apparent viscosity.

**Fig 3:**
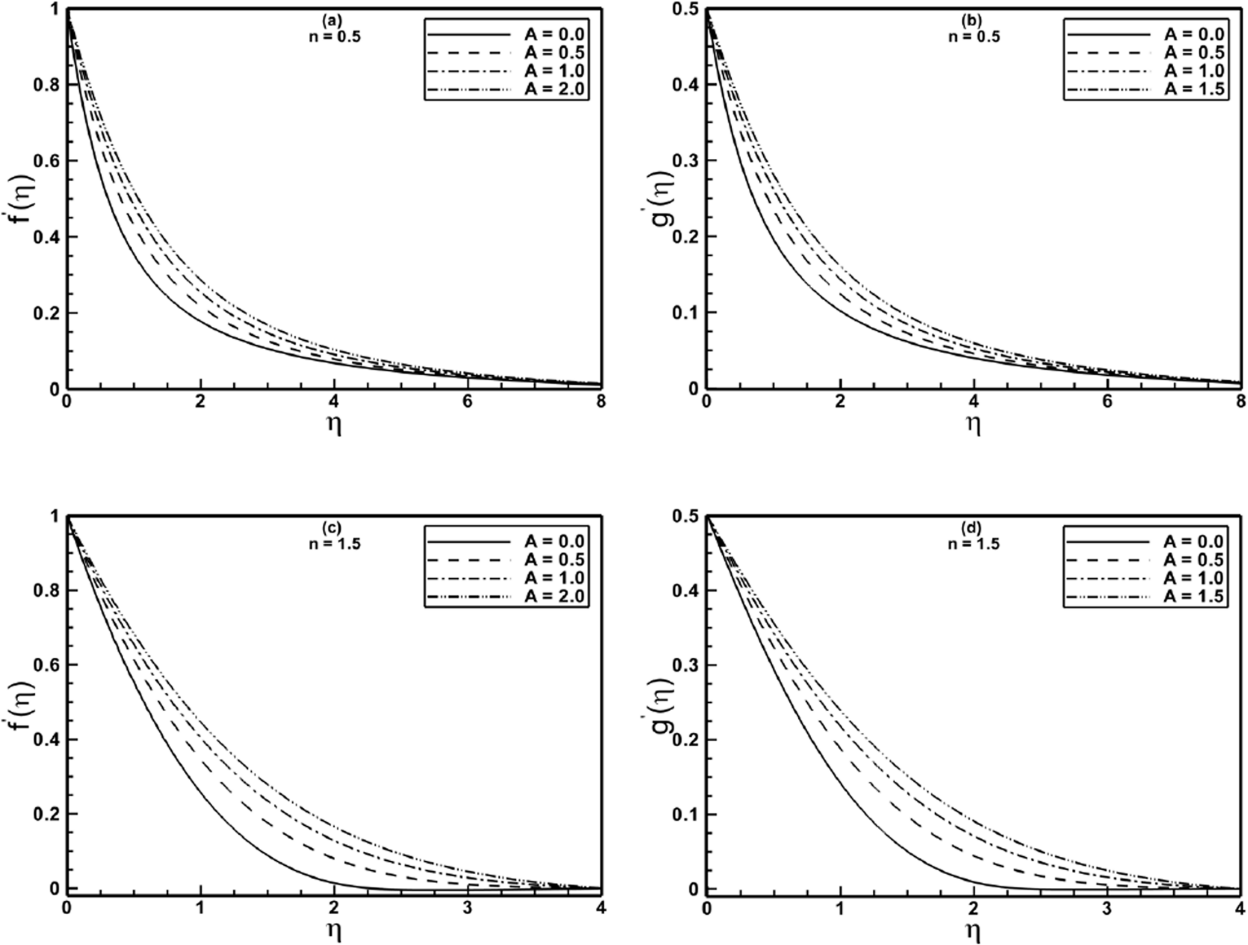
Profiles of the velocities *f*′(*η*) and *g*′(*η*) for different values of the material parameter *A* when *α* = 0.5 is fixed.

Fig 3A–3D depict how the material parameter *A* affects the velocity profiles *f*′(*η*) and *g*′(*η*) for Sisko fluid with shear thinning and shear thickening properties. Fig 3A and 3B show that the velocities profiles increases monotonically with each increment of *A*. The rest of the figures show the same qualitative behavior. Again, Fig 3C and 3D reveal that there is marked decrease in the boundary layer thickness for shear thickening fluids.

**Fig 4:**
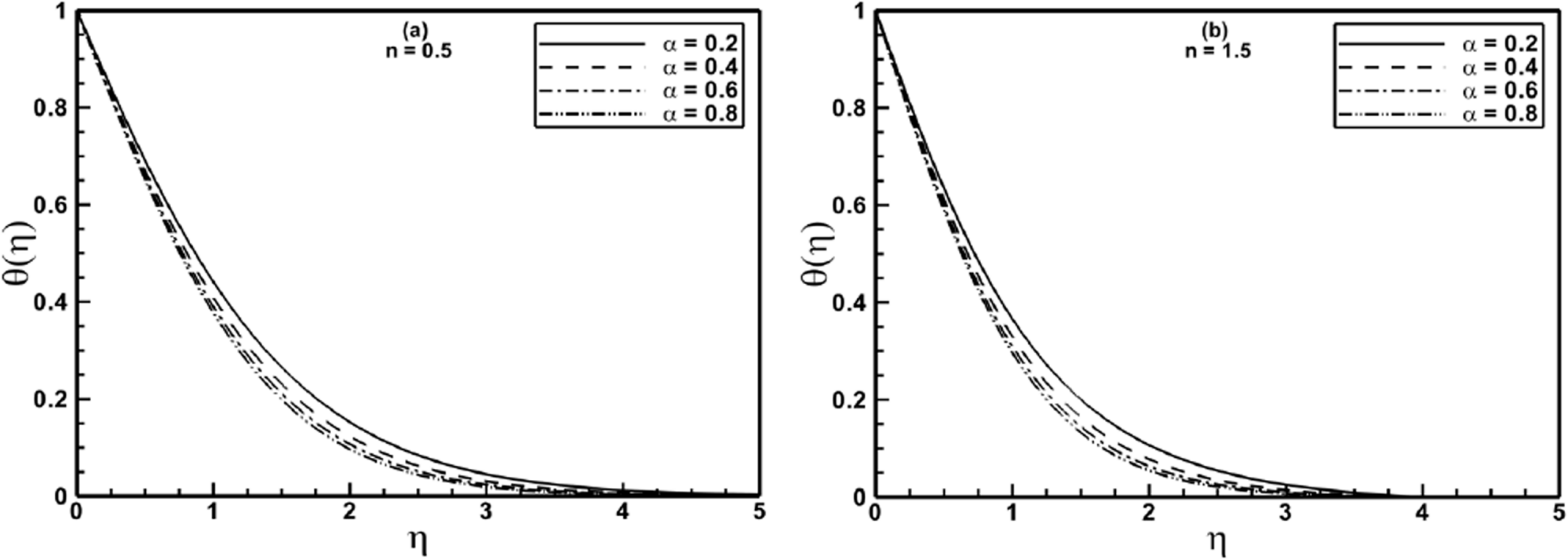
Profiles of the temperature *θ*(*η*) for different values of the stretching ratio parameter *α* when *A* = 1.5 and Pr = 1.0 are fixed.

Fig 4A and 4B depict the influence of stretching ratio parameter *α* on dimensionless temperature *θ*(*η*). From these figures, decrease in dimensionless temperature *θ*(*η*) is noticed for each increment in value of *α*. The reduction in temperature can be attributed to the increased entrainment process. As the value of *α* is incremented, the entrainment of ambient fluid increases toward the isothermal stretching surface, eventually the local fluid temperature decreases. The effect is more noticeable for shear thinning fluid (Fig 4A). Further, it is observed that the thermal boundary layer thickness decreases for shear thickening (*n = 1*.*5*) fluid.

**Fig 5:**
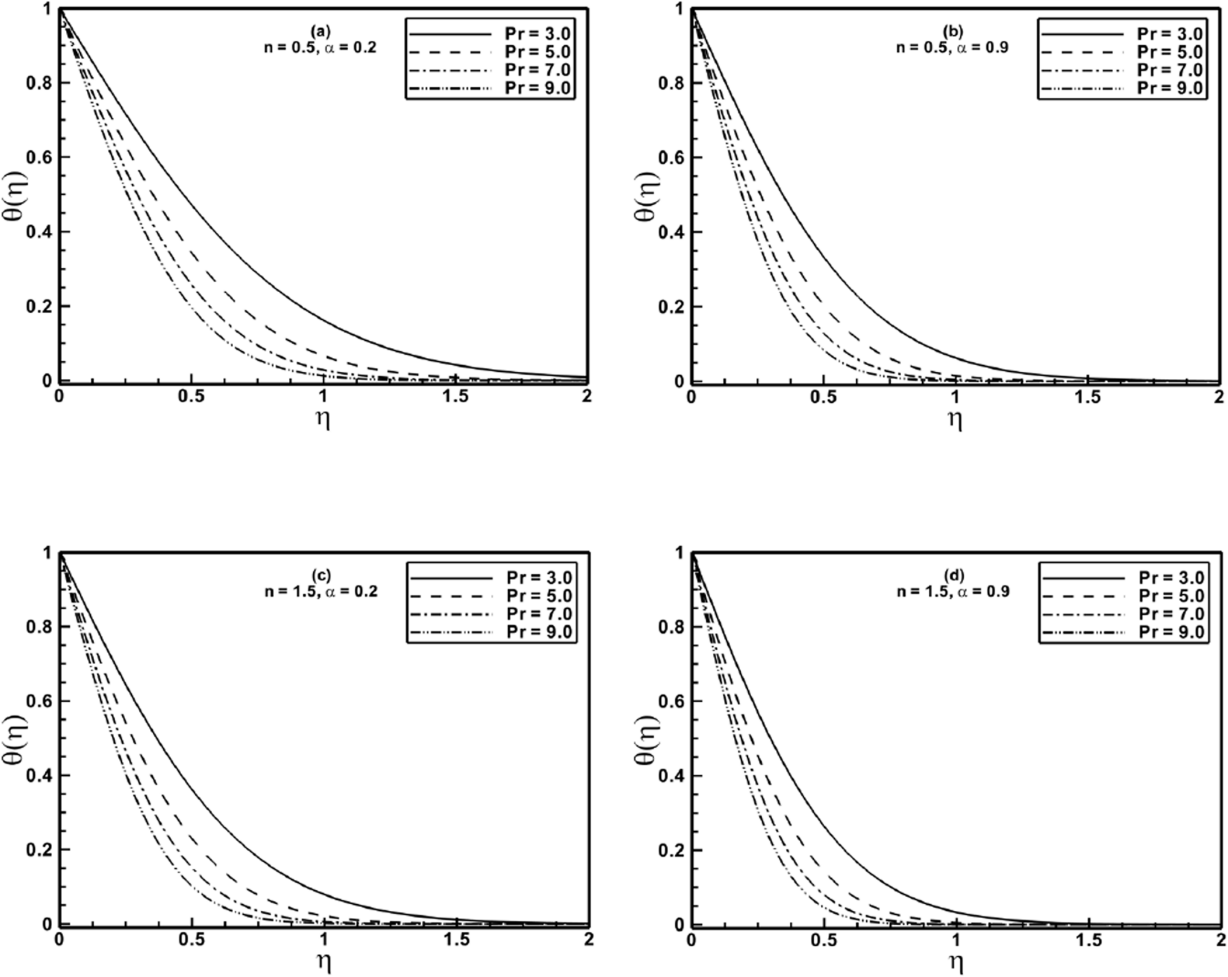
Profiles of the temperature *θ*(*η*) for different values of the Prandtl number Pr when *A* = 1.5 is fixed.

The following figures of temperature profiles are plotted and discussed for selected values of stretching ratio parameter *α*, the Prandtl number Pr and the material parameter *A* of the Sisko fluid. Fig 5A–5D elucidate the effect of the Prandtl number Pr on temperature profile *θ*(*η*) for fixed values of the power-law index *n* and the stretching ratio parameter *α*. These figures reveal that the temperature profile decreases when Pr is increased. A larger Prandtl number fluid has lesser thermal diffusivity, hence it allows diminished thermal effects to penetrate deeper into the fluid. Further, Fig 5B and 5D exhibit that there is a decrease in thermal boundary layer thickness at higher value of the stretching ratio parameter *α*.

**Fig 6:**
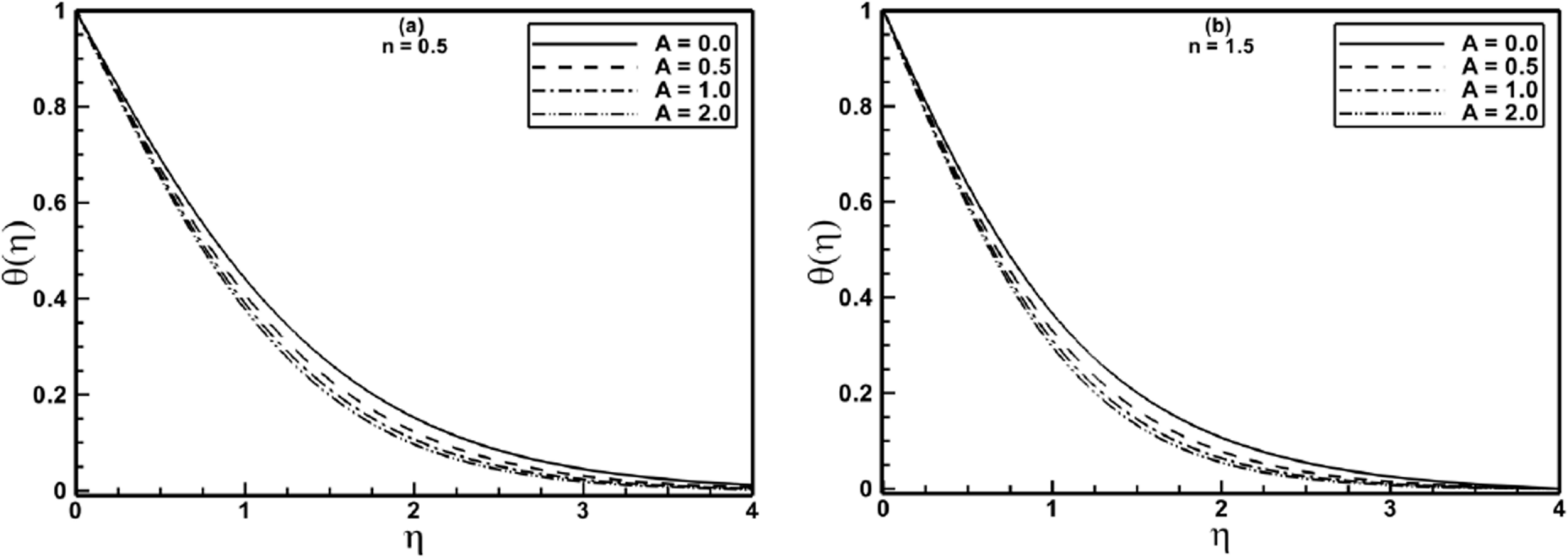
Profiles of the temperature *θ*(*η*) for different values of the material parameter *A* when *α* = 0.5 is fixed.

To exhibit the effects of the material parameter *A* of Sisko fluid on non-dimensional temperature profile, we have plotted Fig 6A and 6B. These plots clearly show that the temperature profile decreases monotonically with increasing values of *A* for the shear thinning and shear thickening fluids. Again, it is noticed that the thermal boundary layer thickness is markedly reduced for shear-thickening (*n = 1*.*5*) fluid. These figures also provide a comparison between the profiles of the power-law fluid (*A = 0*) with those of the Sisko fluid (*A* ≠ 0). From these figures, it is clear that the temperature profiles for the power law fluid is higher when compared with those of Sisko fluid.

**Fig 7:**
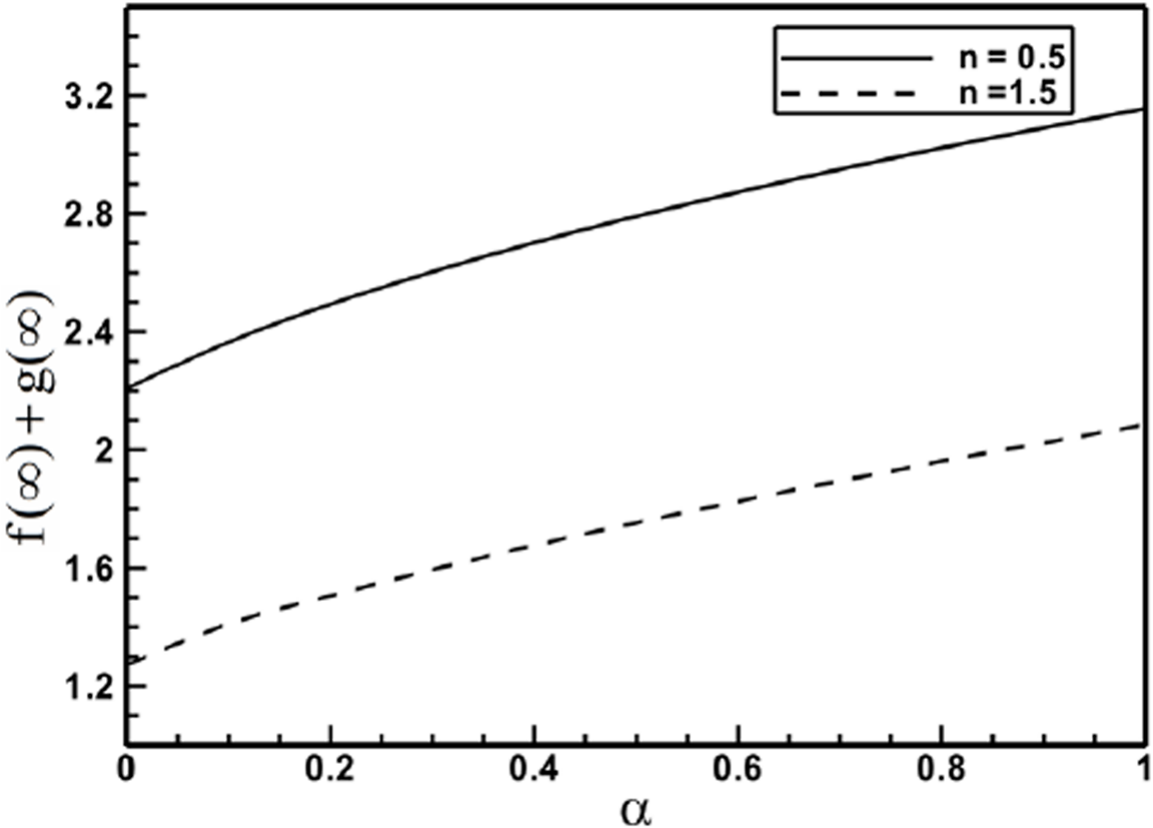
Profiles of the entrainment velocity for different values of the power-law index *n* when *A* = 1.5 is fixed.

Fig 7 graphs the relation between stretching ratio parameter *α* and total entrainment rate, which is proportional to f(∞) + g(∞). This figure depicts that the quantity f(∞) + g(∞) tends to increase monotonically, when *α* is varied from 0 to 1, both for the shear thinning and shear thickening Sisko fluids. The ambient flow rate entrained perpendicular to the stretching sheet is about 43% for shear thinning Sisko fluids, with axis symmetric stretching (*α* = 1) than with unidirectional stretching (*α* = 0). This quantity, however, is 63% higher for shear thickening Sisko fluids.

The magnitude of the numerical values of the skin friction coefficients in the *x* and *y* directions are given in Table 2. It is clear from table that the skin friction drag is higher to some extent for the power-law index less than one. Moreover, it increases with an increase in value of the stretching ratio parameter *α*. Further, it is apparent that the increase is more rapid for the power-law index *n* > 1. Table 3 presents the variation in wall temperature gradient −*θ*′(*η*) with the stretching ratio parameter *α*. It is observed that the value of −*θ*′(*η*) increases with each increment of *α*. This table depicts that the wall temperature gradient is higher for fluid with shear thickening properties and hence results in a higher heat transfer coefficient.

**Table 2:** A tabulation of the local skin friction coefficients along the *x-* and *y-* directions when *A* = 1.5 is fixed.

	*n* = 0.5		*n* = 1.5	
*α*	_$\frac{1}{2}{\text{Re}}_{b}^{1/(n+1)}C_{fx}$_	$\frac{1}{2}{\text{Re}}_{b}^{1/\left(n+1\right)}C_{fy}\left(\frac{U_{w}}{V_{w}}\right)$	$\frac{1}{2}{\text{Re}}_{b}^{1/(n+1)}C_{fx}$	$\frac{1}{2}{\text{Re}}_{b}^{1/\left(n+1\right)}C_{fy}\left(\frac{U_{w}}{V_{w}}\right)$
0.2	-1.74610	-0.24536	-1.60218	-0.23549
0.4	-1.79819	-0.57842	-1.66050	-0.55234
0.6	-1.84739	-0.97856	-1.71569	-0.93386
0.8	-1.89434	-1.43463	-1.76846	-1.37047

**Table 3:** A tabulation of the local Nusselt number when Pr = 1.0 is fixed.

${\text{Re}}_{b}^{-1/(n+1)}\;Nu_{x}$		
*α*	*n* = 0.5	*n* = 1.5
0.2	-0.62074	-0.78919
0.4	-0.69468	-0.84864
0.6	-0.75957	-0.90287
0.8	-0.81827	-0.95324

## Conclusions

The steady three-dimensional flow and heat transfer characteristics within boundary layer of Sisko fluid past an isothermal bidirectional stretching surface have been studied numerically. The effects of the stretching ratio parameter, the material parameter and the Prandtl number on the velocity and temperature profiles were studied. Our computations have indicated that:

- A qualitatively opposite trend was observed in the velocity components *f*′(*η*) and *g*′(*η*) for increasing value of the stretching ratio parameter.
- A substantial reduction in the momentum and thermal boundary layer thickness was noticed for shear thickening fluid.
- Thinning of the thermal boundary layer was seen for increasing Prandtl number and stretching ratio parameter and hence resulted in better heat transfer at the wall.
- The skin friction drag and wall temperature gradient were increased with the stretching ratio parameter.
- The entrainment of ambient fluid, towards the heated plate was much larger for shear thinning Sisko fluids as compared to that of shear thickening Sisko fluids.
